# Skeletal muscle and intermuscular adipose tissue gene expression profiling identifies new biomarkers with prognostic significance for insulin resistance progression and intervention response

**DOI:** 10.1007/s00125-023-05874-y

**Published:** 2023-02-15

**Authors:** Dominik Lutter, Stephan Sachs, Marc Walter, Anna Kerege, Leigh Perreault, Darcy E. Kahn, Amare D. Wolide, Maximilian Kleinert, Bryan C. Bergman, Susanna M. Hofmann

**Affiliations:** 1 Computational Discovery Research, Institute for Diabetes and Obesity (IDO), Helmholtz Diabetes Center (HDC), Helmholtz Zentrum München – German Research Center for Environmental Health, Neuherberg, Germany; 2 German Center for Diabetes Research (DZD), Neuherberg, Germany; 3 Institute for Diabetes and Regeneration (IDR-H), Helmholtz Zentrum München – German Research Center for Environmental Health, Neuherberg, Germany; 4 University of Colorado Anschutz Medical Campus, Aurora, CO USA; 5 Division of Metabolic Diseases, Department of Medicine, Technische Universität München (TUM), Munich, Germany; 6 Drug Development Unit, Institute for Diabetes and Obesity (IDO), Helmholtz Diabetes Center (HDC), Helmholtz Zentrum München – German Research Center for Environmental Health, Neuherberg, Germany; 7 Group of Muscle Physiology and Metabolism, German Institute of Human Nutrition, Potsdam-Rehbruecke (DIfE), Nuthetal, Germany; 8 Department of Medicine IV, University Hospital, LMU Munich, Munich, Germany

**Keywords:** Computational health, Diabetes subtypes, Glucose intolerance, Insulin resistance, Intermuscular adipose tissue, Obesity, Personalised medicine, Response to treatment prediction, Type 2 diabetes

## Abstract

**Aims/hypothesis:**

Although insulin resistance often leads to type 2 diabetes mellitus, its early stages are often unrecognised, thus reducing the probability of successful prevention and intervention. Moreover, treatment efficacy is affected by the genetics of the individual. We used gene expression profiles from a cross-sectional study to identify potential candidate genes for the prediction of diabetes risk and intervention response.

**Methods:**

Using a multivariate regression model, we linked gene expression profiles of human skeletal muscle and intermuscular adipose tissue (IMAT) to fasting glucose levels and glucose infusion rate. Based on the expression patterns of the top predictive genes, we characterised and compared individual gene expression with clinical classifications using *k*-nearest neighbour clustering. The predictive potential of the candidate genes identified was validated using muscle gene expression data from a longitudinal intervention study.

**Results:**

We found that genes with a strong association with clinical measures clustered into three distinct expression patterns. Their predictive values for insulin resistance varied substantially between skeletal muscle and IMAT. Moreover, we discovered that individual gene expression-based classifications may differ from classifications based predominantly on clinical variables, indicating that participant stratification may be imprecise if only clinical variables are used for classification. Of the 15 top candidate genes, *ST3GAL2*, *AASS*, *ARF1* and the transcription factor *SIN3A* are novel candidates for predicting a refined diabetes risk and intervention response.

**Conclusion/interpretation:**

Our results confirm that disease progression and successful intervention depend on individual gene expression states. We anticipate that our findings may lead to a better understanding and prediction of individual diabetes risk and may help to develop individualised intervention strategies.

**Graphical abstract:**

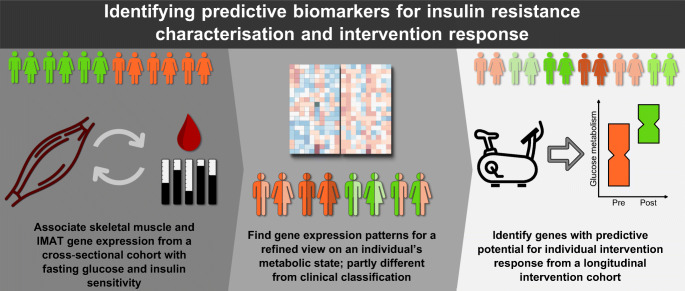

**Supplementary Information:**

The online version of this article (10.1007/s00125-023-05874-y) contains peer-reviewed but unedited supplementary material.

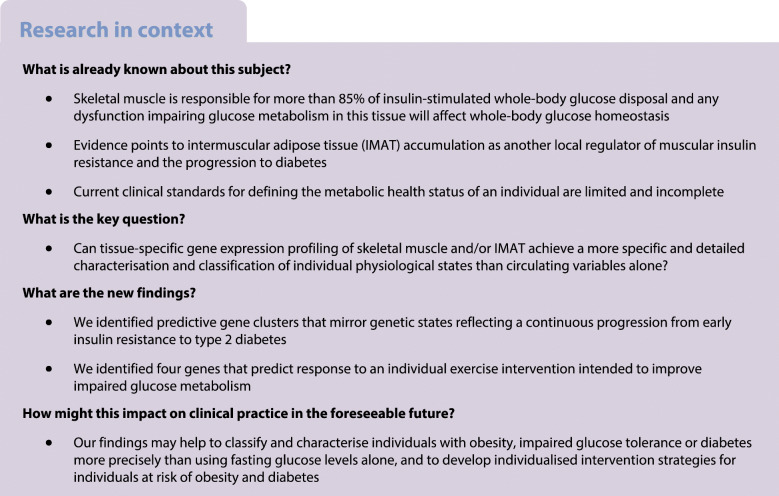



## Introduction

Obesity is a frequent precondition for the development of chronic metabolic diseases such as insulin resistance and type 2 diabetes. Based on the recently published results from the 2017–2018 National Health and Nutrition Examination Survey (NHANES), 42.5% of US adults are currently obese and are thus at high risk for developing type 2 diabetes and its complications [1]. Moreover, the IDF predicts that there will be a 51% increase in the number of individuals with diabetes worldwide by 2045, from 463 million to 700 million, and indicates that one in two adults with diabetes remain undiagnosed at presence [2]. Although the current assessment of diabetes and impaired glucose tolerance is based on purely glycaemic indicators, it is important to emphasise that the risk for developing diabetes is also dependent on age, sex, fat tissue distribution, genetics and gene expression, ethnicity and environmental characteristics. Depending on these individual risk factors and on the inclusion criteria for the cohorts studied, wide heterogeneity in the progression from impaired glucose tolerance to diabetes has been observed. Emerging evidence from a population-based study with 381,363 participants indicates that even people referred to as having ‘metabolically healthy obesity’ are at a substantially higher risk of developing diabetes and its complications [3]. Although medical interventions or changes in lifestyle (diet, exercise) reduce the risk of severe complications, evidence is emerging in population-based cohorts that treatment efficacy also depends on individual genetics [4–7]. This means that patients treated with glucose-lowering interventions will vary in their response, with some gaining a considerable benefit, others seeing no benefit and some experiencing limiting side effects. Taking all of the evidence together, it is becoming increasingly clear that the current clinical standards for defining the metabolic health status of an individual are not adequate and that new strategies for the effective prevention of diabetes are critically important to reduce the burden of this disease. A deeper understanding of the individual features and precise phenotyping of impaired glucose tolerance may improve stratification of disease risk and optimise the benefit/risk ratio and cost-effectiveness of therapeutic approaches for the prevention and treatment of type 2 diabetes.

Given that skeletal muscle is responsible for more than 85% of insulin-stimulated whole-body glucose disposal [8], and that any dysfunction impairing glucose metabolism in this tissue will affect whole-body glucose homeostasis, ultimately contributing to the development of diabetes [9], mechanistic studies mostly focus on this tissue in attempts to elucidate mechanisms involved in metabolic adaptation and its regulation. More recently, evidence has pointed to intermuscular adipose tissue (IMAT) accumulation as another local regulator of muscular insulin resistance and the progression to diabetes [10, 11]. In this study we hypothesised that tissue-specific gene expression profiling of skeletal muscle and/or IMAT could achieve a more specific and detailed characterisation and classification of individual physiological states than circulating variables alone. We further presumed that the expression of individual genes might (1) allow the prediction of individual disease-related states; (2) identify individuals with a high or low risk for diabetes; and (3) enable the potential response of a given individual to a specific treatment strategy to be predicted.

To this end, we aimed to investigate dependencies between gene expression in skeletal muscle and/or IMAT and clinical diabetes markers from individuals with obesity, with and without type 2 diabetes. We used multivariate regression to model the tissue-specific gene expression impact on the two key insulin resistance markers, glucose infusion rate (GIR) during a hyperinsulinaemic–euglycaemic clamp and fasting glucose (FG). We used a clustering approach to compare states (obesity and type 2 diabetes) defined by metabolic-related gene expression patterns with binary clinical classifications. Finally, we tested selected genes for their potential to predict individual intervention response based on an independent lifestyle and exercise intervention study.

## Methods

### Human transcriptional profiling dataset

**Fig. 1:**
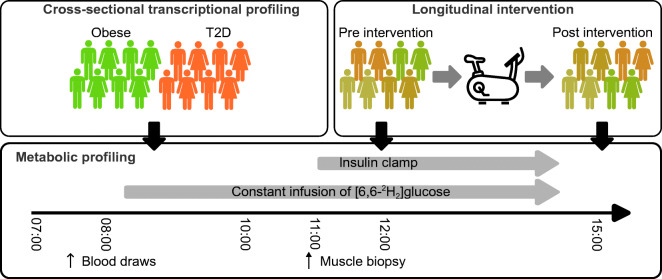
Study designs. Data for cross-sectional transcriptional profiling were obtained from a cohort of individuals with obesity and type 2 diabetes (T2D). Intervention study data were collected from individuals with obesity, with and without impaired glucose tolerance and impaired FG, pre and post exercise intervention. The design of the metabolic profiling, including the hyperinsulinaemic–euglycaemic clamp, was identical for both studies

Human muscle and IMAT transcriptional profiles were obtained from a cross-sectional study previously reported by Sachs et al [12] (Fig. 1). To identify features suitable for characterisation of individual prediabetic states and potentially predictive for disease progression we selected all 16 participants with obesity and type 2 diabetes for whom paired samples were available. All participants were clinically characterised by determining age, BMI, body weight, FG, fasting insulin, fat-free mass, glucagon, height, insulin sensitivity via GIR during a hyperinsulinaemic–euglycaemic clamp and relative fat mass (Table 1). In total, 13 participants were of white ethnicity and three were of Hispanic ethnicity.

**Table 1:** Participant demographics: human transcriptional profiling (*n=*16)

Clinical variable	Obesity	Type 2 diabetes
No. of participants	10	6
Age (years)	40.5 ± 2.4	45.7 ± 2.5
BMI (kg/m^2^)	36.7 ± 1.6	34.8 ± 1.7
BW (kg)	116.8 ± 7.4	101.4 ± 6.4
FFM (kg)	71.9 ± 4.8	63.0 ± 4.5
FG (mmol/l)	4.9 ± 0.2	10.2 ± 0.9***
Fasting insulin (pmol/l)	122.2 ± 22.9	173.6 ± 27.8
Glucagon (ng/l)	70.6 ± 9.7	87.7 ± 10.1
Height (m)	1.8 ± 0.04	1.7 ± 0.03
Insulin sensitivity/GIR (mg kg^–1^ min^–1^)	5.1 ± 1.0	1.7 ± 0.7*
RelFat (%)	38.2 ± 2.6	37.8 ± 2.7

Data are mean ± SEM

The human transcriptional profiling dataset included eight participants of white ethnicity and two participants of Hispanic ethnicity with obesity, and five participants of white ethnicity and one of Hispanic ethnicity with type 2 diabetes. Insulin sensitivity data were normalised to kg of BW

*p<0.05 and ***p<0.001 for difference between obesity and type 2 diabetes (one-way ANOVA)

### Longitudinal intervention dataset

Seventeen individuals with obesity (BMI 30–40 kg/m^2^), with and without impaired glucose tolerance and impaired FG, were recruited for this study from the local Denver area. Impaired FG was defined as FG between 5.6 and 7 mmol/l, with postprandial glucose <7.8 mmol/l 2 h after a 75g OGTT. Impaired glucose tolerance was defined as normal FG <5.6 mmol/l, with postprandial glucose >7.8 mmol/l 2 h after a 75g OGTT. A list of participant exclusion criteria is available in the electronic supplementary material (ESM; see ‘Longitudinal intervention study’). Participants were asked to refrain from planned physical activity for 48 h before the first and second metabolic studies and were given a standardised diet for 7 days prior to each study (Fig. 1). After overnight fasting a basal muscle biopsy was taken; this was followed by metabolic profiling including a 3 h hyperinsulinaemic–euglycaemic clamp. After the first metabolic study, participants entered a 12 week supervised weight loss and exercise training intervention. The weight loss intervention consisted of a medically supervised low energy diet comprising a meal replacement product that can be consumed as a liquid or made into a variety of food forms (Health & Nutrition Technology, Carmel, CA, USA). Participants were provided with powdered Health One formula and instructed to consume five portions per day, providing 3724 kJ/day (890 kcal/day), 75 g of protein, 15 g of fat and 110 g of carbohydrate and 100% of the daily recommended intake of all vitamins, minerals and micronutrients. The exercise training consisted of four individually supervised sessions per week of whole-body aerobic activity. During the first 2–3 weeks of training, the exercise duration and intensity were gradually increased to 45 min at 80–85% of the maximal heart rate. After the 3 month intervention, participants transitioned to a 2 week weight maintenance diet. Participants continued to exercise during the weight stabilisation period. After completing the intervention and 2 week weight maintenance period, participants then repeated the metabolic study with the muscle biopsy and 3 h hyperinsulinaemic–euglycaemic clamp (Table 2). Metabolic studies consisted of measurements of age, BMI, BW, FG, fasting insulin, FFM, glucagon, insulin sensitivity via GIR during the hyperinsulinaemic–euglycaemic clamp, relative fat mass and $\dot{V}{\mathrm{O}}_{2\mathrm{peak}}$ (Table 2). See ESM, ‘Longitudinal intervention study’, for further details.

**Table 2:** Participant demographics: longitudinal intervention study (*n*=17)

Clinical variable	Pre intervention	Post intervention
Age (years)	46.5 ± 2.2	
BMI (kg/m^2^)	34.7 ± 1.0	30.7 ± 1.0***
BW (kg)	96.9 ± 2.7	85.9 ± 2.6***
FFM (kg)	56.7 ± 1.8	52.9 ± 1.5***
FG (mmol/l)	5.2 ± 0.1	5.0 ± 0.1
Fasting insulin (pmol/l)	110.4 ± 9.7	78.5 ± 9.7***
Glucagon (ng/l)	82.2 ± 4.0	71.4 ± 3.7***
Insulin sensitivity/GIR (mg kg^–1^ min^–1^)	3.5 ± 0.4	5.4 ± 0.5***
RelFat (%)	41.3 ± 1.5	38.0 ± 1.8***
$\dot{V}{\mathrm{O}}_{2\mathrm{peak}}$ (l/min)	2.2 ± 0.1	2.5 ± 0.1*
Relative $\dot{V}{\mathrm{O}}_{2\mathrm{peak}}$ (ml/kg min^–1^)	23.2 ± 1.0	29.6 ± 1.4*

Data are mean ± SEM

The study included 12 individuals of white, three of Hispanic, one of East Indian and one of African American ethnicity. Ethnicity was not taken into account in statistical analyses. Insulin sensitivity data were normalised to kg of BW

*p<0.05 and ***p<0.001 for difference from pre intervention (paired *t* test)

Changes in metabolic variables pre to post intervention were estimated using a paired *t* test. Pre- and post-intervention biopsies were used for gene expression analysis. Because insufficient RNA was isolated from the IMAT samples or the RNA integrity number did not match quality requirements for gene expression analysis, we removed all IMAT samples and used only the remaining muscle samples for gene expression analysis. Pre- to post-differential gene expression was estimated using one-way ANOVA. See ESM, ‘Gene expression analysis’, for further information.

### Models and statistics

To estimate the impact of gene expression on FG and GIR we used linear multivariate regression models. Thus, we created one predictive model for each gene, simultaneously predicting clinical variables based on gene expression in skeletal muscle and IMAT. Models can be formalised in matrix notation as **Y** = β**X**_**j**_ + ε, where **Y** is a matrix of the sampled response variables GIR (g) and FG (f) and **X** is a matrix of the predictor values, the expression of gene j in muscle (m) and IMAT (i). β forms the 2 × 2 matrix of the four estimated regression coefficients β_mg_, β_mf_, β_ig_ and β_if_ describing the four relationships between tissue-specific gene expression and response variables. The residues or errors are formed in ε. Our approach can be interpreted as a mixture model that allows us to jointly estimate these four coefficients in one model to predict insulin sensitivity and glucose homeostasis from gene expression in muscle and IMAT. Genes that contributed the most to insulin sensitivity and glucose homeostasis were then scored based on the log-likelihood and negative log-likelihood of the single regression models. Subsequently, hierarchical clustering was performed to group the selected genes into distinct clusters with similar expression profiles. These clusters were then used to compare gene expression profiles with diagnosed disease states by generating participant *k*-nearest neighbour (kNN)-networks with *k*=3 nearest neighbours, using the Euclidean distance metric. The predictive classification score was calculated from the maximum ratio of each participant’s direct neighbour’s clinical classification (percentage with obesity vs percentage with type 2 diabetes). Analysis was carried out using Matlab R2020a (https://www.mathworks.com). See ESM for further information.

### Transcriptomic profiling

Skeletal muscle and IMAT samples from the cross-sectional study were used for transcriptional profiling. See ESM for further information.

### Gene expression analysis

Quantitative reverse transcription PCR (qRT-PCR) was used to determine relative mRNA expression levels. See ESM for further information.

## Results

### Multivariate regression unravels tissue-specific gene expression patterns correlating with insulin resistance

**Fig. 2:**
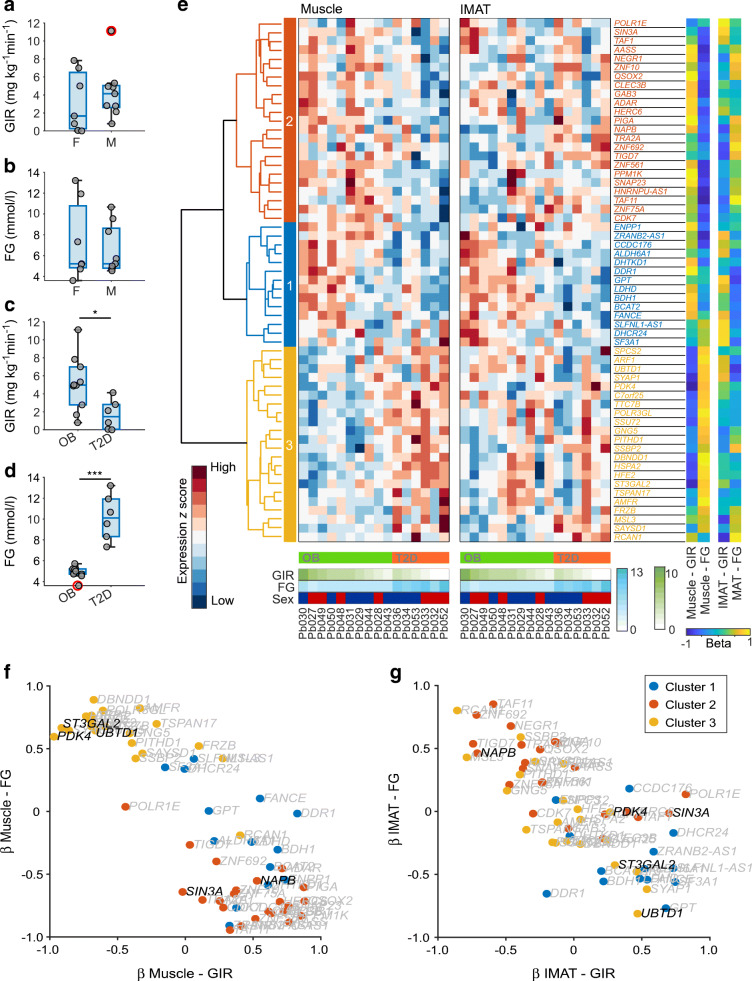
Multivariate regression analysis unravels tissue-specific gene expression patterns correlating with insulin resistance. (**a**–**d**) Boxplots comparing FG (mmol/l) and insulin sensitivity (GIR, mg kg^–1^ min^–1^) distributions between participants of different sexes (**a**, **b**) and classifications (**c**, **d**). F, female; M, male; OB, obesity; T2D, type 2 diabetes. Red circles indicate outliers. *p<0.05 and ***p<0.001 (one-way ANOVA). (**e**) Heatmaps of muscle and IMAT genes correlating with insulin resistance identified by multivariate regression. Colours in the dendrogram refer to clusters 1 (blue), 2 (red) and 3 (yellow). The colours in the bars below the heatmaps indicate individual disease classification, GIR, FG levels and sex. Vertical colour bars show the four estimated regression coefficients for each gene, indicating the four relationships between tissues and response variables (GIR and FG). (**f**, **g**) Scatterplots comparing gene-specific β coefficients for GIR and FG for muscle (**f**) and IMAT (**g**). The colours of the dots refer to gene cluster assignment

We first compared participants’ demographic and metabolic variables. As expected, we found sex-specific differences in RelFat (*p*<0.05), FFM (*p*<0.001) and height (*p*<0.01) (ESM Fig. 1a). We also found significant differences in GIR (*p*<0.05) and FG (*p*<0.001) between participants with obesity and those with type 2 diabetes (Table 1, Fig. 2c,d). Additionally, as expected, we found that low GIR values correlate with high FG levels (ESM Fig. 1b). As shown in Fig. 2c,d, individuals with obesity (BMI >30 kg/m^2^ and FG <7 mmol/l) exhibited a wide range of insulin sensitivities (GIR 0.8–11.1 mg kg^–1^ min^–1^). Some individuals with more severe insulin resistance (GIR <3 mg kg^–1^ min^–1^) were still able to maintain FG levels at <7 mmol/l. In contrast, some individuals with diabetes exhibited a better GIR, with levels up to 4 mg kg^–1^ min^–1^. To explore whether transcriptional changes in muscle and IMAT at the transition from obesity with compensated insulin resistance (normoglycaemia) to type 2 diabetes (hyperglycaemia) reflect the inconsistency between FG and GIR, we performed a multivariate regression analysis to identify genes whose expression had a strong link to insulin resistance and glucose homeostasis.

After multivariate regression analysis we selected the top 59 genes (ESM Fig. 1c) contributing to GIR and FG and used *k*-means to cluster them into three clusters of 14 (cluster 1), 23 (cluster 2) and 22 (cluster 3) genes with distinct expression patterns in muscle and IMAT (Fig. 2e, ESM Fig. 1d, ESM Table 1). We compared the corresponding β values of the gene clusters and observed three distinct patterns (Fig. 2f,g, ESM Fig. 1e). Cluster 1 included genes whose expression was positively associated with GIR and negatively associated with FG in both muscle and IMAT, thus correlating with healthy glucose metabolism. Cluster 2 contained genes whose expression was positively correlated with GIR and negatively correlated with FG in muscle, similar to cluster 1, with opposing associations for most of the observed genes in IMAT. Cluster 3, on the contrary, contained genes whose expression was negatively correlated with GIR and positively correlated with FG in muscle, with no effect observed in IMAT.

Our results suggest that these largely different gene expression profiles in muscle and IMAT are associated with varying impacts on glucose metabolism. In particular, expression of *PDK4*, which has been linked to diabetes and glucose metabolism previously [13], shows a high correlation with GIR and FG in muscle but almost none in IMAT (Fig. 2f,g). We also identified genes with opposing effects on glucose metabolism in muscle compared with IMAT, such as *UBTD1* and *ST3GAL2*. Both genes show strong positive β coefficients for FG and negative β coefficients for GIR in muscle whereas in IMAT we observed negative β coefficients for FG and positive β coefficients for GIR (Fig. 2f,g). Both genes were associated with cluster 3. In contrast, *NAPB* from cluster 2 shows the opposite associations. In a third observation we found genes, here represented by *SIN3A*, that seem to have a relatively high predictive value for FG but a low or no predictive value for GIR in muscle but completely opposing values in IMAT (high value for GIR, low value for FG).

Taken together, the genes identified in all three clusters show a striking association between expression in muscle and GIR and FG, while only genes in cluster 1 show an association between IMAT expression and glucose homeostasis and insulin sensitivity (Fig. 2f,g, ESM Fig. 1e). These results suggest that muscle gene expression profiles may allow for a more specific and detailed characterisation and classification of individual physiological states than serum-based physiological variables alone.

### Gene expression-based classification enables a refined view of the individual physiological state of individuals with obesity

**Fig. 3:**
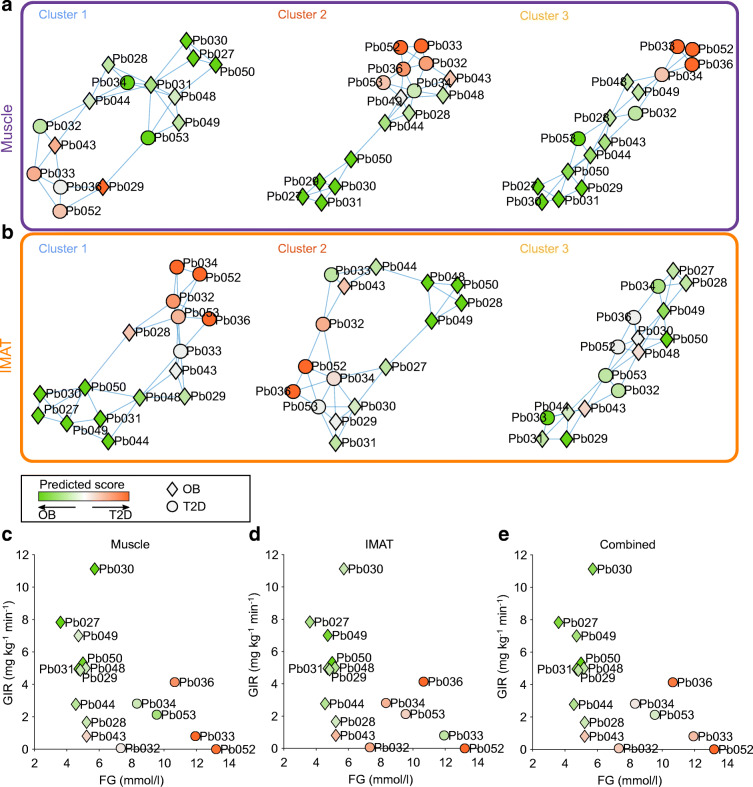
Gene expression-based participant classification reveals a refined view of physiological state. (**a**, **b**) kNN-networks for the three clusters in muscle (**a**) and IMAT (**b**). Nodes refer to individual participants. Node shape refers to the assigned clinical classification: obesity (OB; diamond) or type 2 diabetes (T2D; circle). Node colour refers to the estimated disease state based on connected individuals. (**c**–**e**) Scatter plots displaying the clinical variables FG and GIR for all individuals. Node shape refers to the assigned clinical classification: OB (diamond) or T2D (circle). Node colour refers to the estimated disease state across all three gene clusters for muscle (**c**), IMAT (**d**) and both tissues combined (**e**)

To test our hypothesis that gene expression patterns are superior to conventional clinical markers for categorising individual insulin resistance states, we performed a kNN classification for each tissue and gene cluster based solely on expression profiles. Thus, we generated six nearest neighbour networks (NNNs) representing expression-based participant similarities for all 16 participants (Fig. 3a,b). Based on direct network neighbours we then calculated a predictive classification score for each individual (Fig. 3a,b, ESM Fig. 2). For muscle, we found a non-unique classification over all three NNNs for five of the 16 participants (two with obesity: Pb029, Pb043; three with type 2 diabetes: Pb034, PB053, Pb032). After averaging over the three clusters, one participant with obesity was classified as having type 2 diabetes (Pb043) and two participants with type 2 diabetes were classified with obesity (Pb034, Pb053). For the IMAT-derived NNNs we found a predicted classification that differed from the clinical classification for seven participants (three with obesity: Pb048, Pb028, Pb043; four with type 2 diabetes: Pb034, Pb053, Pb033, Pb032). Averaging over the three clusters resulted in a different classification for two participants compared with the clinical classification: Pb043 (obesity) and Pb033 (type 2 diabetes). Averaging over both tissues, we identified two participants with a divergent classification: Pb043 and Pb053. Overall, the classification of obesity was more consistent than the classification of type 2 diabetes, with only two participants consistently classified as having type 2 diabetes (Pb036, Pb052).

When comparing the NNN-based classification with metabolic variables, we found that an increase in the probability of developing type 2 diabetes correlated with decreasing GIR for participants with obesity in both muscle tissue and IMAT (Fig. 3c–e). In contrast, for hyperglycaemic participants clinically classified as having type 2 diabetes (FG >7 mmol/l), the NNN-based classification did not correlate with either GIR or FG in either tissue. These results suggest that, beyond a binary clinical classification of type 2 diabetes and normoglycaemia as FG >7 mmol/l and FG ≤7 mmol/l, respectively, there is a continuous development from insulin resistance to diabetes that follows an individual trajectory, which means that there is diagnostic potential to predict an individual’s risk for diabetes or their potential to respond to interventions.

### *ST3GAL2*, *SIN3A*, *ARF1* and *AASS* mRNA levels in muscle tissue predict intervention response

**Fig. 4:**
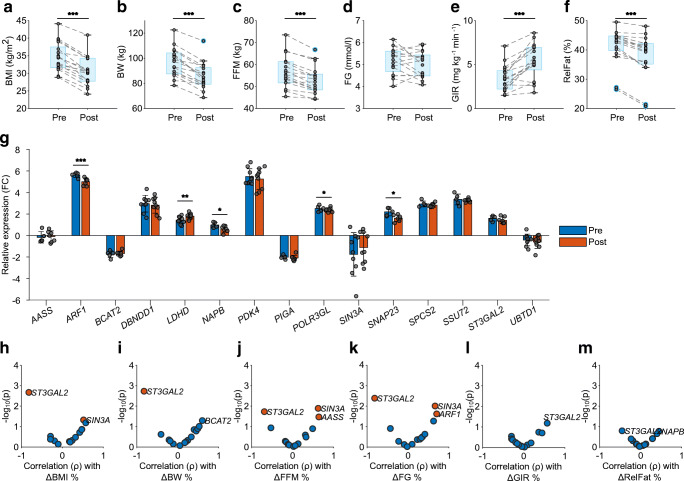
*ST3GAL2*, *SIN3A*, *ARF1* and *AASS* mRNA expression in muscle predicts intervention response. (**a**–**f**) Changes in clinical variables from pre to post intervention: (**a**) BMI, (**b**) BW, (**c**) FFM, (**d**) FG, (**e**) GIR and (**f**) RelFat. ***p<0.001 (paired *t* test). (**g**) Relative mRNA levels (to the reference gene *TBP*) of selected genes pre and post intervention, shown as log_2_ (${2}^{-\Delta \Delta {\mathrm{C}}_{\mathrm{t}}}$). *p<0.05, **p<0.01 and ***p<0.001 (one-way ANOVA). Error bars denote SEMs. (**h**–**m**) Correlation volcano plots of pre-intervention mRNA expression and relative change in clinical variables between pre and post intervention: (**h**) BMI (kg/km^2^), (**i**) BW (kg), (**j**) FFM (kg), (**k**) FG (mmol/l), (**l**) GIR (mg kg^–1^ min^–1^) and (**m**) RelFat (%). Significantly correlated mRNAs are shown in orange. FC, fold change

To evaluate whether gene expression profiles within muscle and/or IMAT define individual health states with predictive potential for disease progression or modulation of insulin sensitivity, we analysed 17 individuals with obesity, with and without impaired glucose tolerance, undergoing a combined weight loss and exercise training intervention (Table 2). Clinical variables such as GIR, FG, BW, RelFat, FFM and BMI were measured pre and post intervention. Almost all individuals showed an increase in GIR (*p*=2.2 × 10^–5^) after the intervention and a decrease in BMI (*p*=2.3 × 10^–8^), BW (*p*=6.1 × 10^–8^), RelFat (*p*=2.7 × 10^–6^) and FFM (*p*=1.4 × 10^–6^). A change in FG levels post intervention was not observed (*p*=0.12) (Fig. 4a–f). However, when correlating the relative pre/post change (Δ%) between all clinical variables, we found that a change in GIR was significantly correlated only with a change in BW (ESM Fig. 3). A decrease in FG, in turn, was significantly correlated with a relative decrease in BMI, FFM and BW.

To test if these physiological changes are linked to individual gene expression in muscle, biopsies taken before and after the intervention were used for RNA expression analysis. Six pre- and eight post-intervention muscle samples did not meet quality requirements and were removed from subsequent analyses. We combined various criteria to select 15 candidate genes for validation from the three clusters of 59 genes initially identified in our first patient cohort. Gene expression in muscle tissue was measured using qRT-PCR (ESM Table 2) in this second independent intervention trial. Among the genes selected, *SIN3A*, *UBTD1*, *ST3GAL2* and *NAPB* showed notable β value profiles (Fig. 2f,g). *AASS*, *DBNDD1*, *PDK4*, *PIGA*, *POLR3GL*, *SNAP23*, *SPCS2*, *SSU72* and *UBTD1* could be linked to diabetes-associated SNPs identified in the Type 2 Diabetes Knowledge Portal [14] and *ARF1*, *BCAT2* and *LDHD* could be linked to skeletal muscle lipid and glucose metabolism and insulin resistance [15–17]. *PDK4* was included as a well-described marker of muscle insulin resistance and as a potential therapeutic target [18]. Of these 15 genes, five (*LDHD*, *ARF1 NAPB*, *POLR3GL* and *SNAP23*) showed a significant change in expression between pre and post intervention (Fig. 4g). As we hypothesised that distinct gene expression states may relate to individual disease states, we tested selected genes for their predictive potential for individual intervention response. To this end, we correlated individual pre-intervention gene expression with the relative change (pre to post) in the clinical variables BMI, BW, FFM, FG, GIR and RelFat (Fig. 4a–f). ΔGIR and ΔRelFat could not be significantly correlated with any of the genes tested. A change in the remaining variables could be significantly predicted by the genes *ST3GAL2* (FG, BW, FFM and BMI), *SIN3A* (FG, FFM and BMI), *ARF1* (FG) and *AASS* (FFM) (Fig. 4h–m, ESM Fig. 4, ESM Table 3). In contrast to the five genes that showed a change in expression post intervention (*LDHD*, *ARF1*, *NAPB*, *POLL3GL* and *SNAP23*), none of the four genes identified with predictive character appeared to be differentially expressed between pre and post intervention (Fig. 4g). Together, these findings indicate that individual susceptibility to exercise intervention for the improvement of glucose homeostasis is independent of the individual clinical variables, but correlates with individual gene expression profiles prior to intervention. We next compared these four identified genes with *PDK4*, a well-described muscle marker for insulin resistance. To our surprise, *PDK4* was not significantly associated with any intervention-induced change in metabolic variables (ESM Fig. 4).

Next, we found that low expression levels of three of the four genes (*AASS*, *ARF1* and *SIN3A*) was associated with a good health prognosis. In particular, *ARF1* showed a significant decrease in expression on exercise intervention. In turn, *ST3GAL2* was the only gene for which increased expression levels in muscle tissue increased the likelihood of an effective intervention. In summary, within this independent intervention trial we were able to validate our hypothesis that muscle gene expression profiles have predictive potential for individual insulin resistance states.

## Discussion

In this study we showed that human transcriptional profiles of skeletal muscle and IMAT from individuals with obesity, with and without type 2 diabetes, are differentially coupled to insulin resistance and glucose homeostasis. We identified predictive gene clusters that mirror gene expression states reflecting a continuous progression from early insulin resistance to type 2 diabetes according to individual traits. From a subset, the genes *AASS*, *ARF1*, *SIN3A* and *ST3GAL2* predicted individual improvement of impaired glucose metabolism by means of an exercise and lifestyle intervention.

We started our analysis with the observation that there is overlap of GIR measurements between obesity and type 2 diabetes. We hypothesised that the binary clinical classification of type 2 diabetes does not reflect individual underlying gene expression states and that specific gene expression patterns in skeletal muscle and/or IMAT may have the potential to identify and predict individuals with a high or low risk of developing diabetes or to predict individual susceptibility to interventions.

Our multivariate regression analysis revealed a strong association of all three gene clusters in muscle with GIR and FG, while only the genes in cluster 1 in IMAT were associated with glucose homeostasis and insulin resistance. Together with the observation that β coefficients estimates in cluster 2 showed opposing associations with FG and GIR in muscle compared with IMAT, we concluded that IMAT and muscle contribute differentially to glucose metabolism. However, there is higher variance in IMAT gene expression [12], which may mean that any correlation is harder to detect. The increased variability in IMAT gene expression may arise from technical difficulties in dissecting IMAT from muscle, resulting in less material for RNA extraction, or the higher heterogeneity of IMAT itself, which is composed of multiple cell types such as pre-adipocytes, adipocytes, adipocyte-like cells, myoblasts and stromal and vascular cells.

Participant classification based on kNN-networks revealed that insulin sensitivity could be accurately predicted for individuals without diabetes from gene expression patterns, whereas gene expression patterns for participants with hyperglycaemia scored differently from the clinical classification in several cases. The latter observation is consistent with the idea that hyperglycaemia occurs in a late state of disease progression as a consequence of pancreatic beta cell failure. In addition, insulin sensitivity is associated with multiple organ malfunction and, in particular, skeletal muscle is the primary organ for glucose uptake [19]. Although GIR measured using a hyperinsulinaemic–euglycaemic clamp is still the gold standard for directly measuring insulin resistance, it is highly invasive and time-consuming and has very limited predictive potential. FG levels by themselves are unlikely to identify individuals with obesity and impaired glucose tolerance; rather, they identify individuals with severe insulin resistance with an increased risk for irreversible damage of tissues and organs [20]. We thus conclude that both GIR and FG levels are not suitable for a reliable early diagnosis and prognosis of disease progression. In contrast, the gene expression profiles identified here, which represent a muscle-specific state of individual insulin resistance, have predictive potential for the characterisation of individual insulin sensitivity.

This predictive potential was tested on muscle tissue from an additional independent cohort of 17 individuals with impaired glucose metabolism undergoing a 12 week combined weight loss and exercise intervention. By correlating the pre-intervention expression levels of our candidate genes with the relative change in clinical variables post intervention we identified four genes with significant predictive value: *AASS*, *ARF1*, *SIN3A* and *ST3GAL2*. Lower levels of expression of *AASS*, *ARF1* and *SIN3A* indicated a positive prognosis. *AASS* encodes the enzyme aminoadipate-semialdehyde synthase, which is involved in mammalian lysine degradation and in hyperlysinaemia [21], but which has not yet been characterised in the context of impaired glucose metabolism, insulin resistance or diabetes. Beside its predictive potential we also found that expression of *ARF1*, which encodes ADP ribosylation factor 1, was significantly reduced after the intervention. ADP ribosylation factor 1 was recently linked to rapamycin (mTOR) complex 2 (mTORC2) [22], which has been shown to be involved in exercise-dependent regulation of muscle glucose uptake in mice [23]. SIN3 transcription regulator family member A, encoded by *SIN3A*, has been linked to glucose metabolism in murine beta cells [24]. It has further been shown that SIN3A is an insulin-sensitive forkhead box protein O1 (FOXO1) corepressor of glucokinase in murine liver [25]. SIN3A was also shown to negatively regulate insulin receptor (*Insr*/*INSR*) mRNA in mice and human muscle [26]. Finally, *ST3GAL2*, which encodes ST3 beta-galactoside alpha-2,3-sialyltransferase 2, was the only gene identified to positively predict exercise response at high expression levels. Mice lacking this protein have been shown to develop obesity and insulin resistance after 7–9 months of age [27]. In summary, three of the four predictive genes that we identified have already been linked to insulin resistance and diabetes but their predictive potential has not yet been explored.

In conclusion, we identified novel markers for predicting impaired insulin sensitivity in human muscle and found four markers that predict individual exercise intervention responses in participants with diabetes. These findings may help to classify and characterise individuals with obesity, impaired glucose tolerance or diabetes more precisely than using state-of-the-art variables such as GIR and FG alone. Additionally, we anticipate that these findings may also help to develop precise and individualised intervention strategies for patients at risk of obesity and type 2 diabetes.

## Supplementary information

ESM 1 (PDF 2365 kb)

## Data Availability

Gene expression profiles and clinical data can be shared on request to BCB. Statistical code not provided in the manuscript or supplementary information is available on request to DL

## Abbreviations

BW: Body weight
FFE: Fat-free mass
FG: Fasting glucose
GIR: Glucose infusion rate
IMAT: Intermuscular adipose tissue
kNN: *k*-nearest neighbour
NNN: Nearest neighbour network
qRT-PCR: Quantitative reverse transcription PCR
RelFat: Relative fat mass
