# Supplementary material for: Skeletal muscle and intermuscular adipose tissue gene expression profiling identifies new biomarkers with prognostic significance for insulin resistance progression and intervention response
Source: Diabetologia. 2023 Feb 15;66(5):873–83. doi: 10.1007/s00125-023-05874-y (PMC10036433; doi:10.1007/s00125-023-05874-y)
Supplement: Supplementary file 1 — (PDF 2365 kb) [file 125_2023_5874_MOESM1_ESM.pdf]

ESM Methods

Longitudinal Intervention Study

Cohort description: Obese men and women with pre-diabetes were recruited for this study from the local Denver area with ages ranging from 30-60 yrs. Individuals with impaired fasting glucose (IFG), impaired glucose tolerance (IGT), and a combination of the two (IFG/IGT) were recruited and verified using oral glucose tolerance testing at initial screening. All subjects were be obese (BMI 30-40 kg/m2) and sedentary (<2 hours/week of planned physical activity). Impaired fasting glucose is defined as fasting glucose between 5.6 – 7 mmol/l, with post-prandial glucose (<7.8 mmol/l 2 hrs after 75g OGTT). Impaired glucose tolerance is defined as normal fasting glucose <5.6 mmol/l, with post-prandial glucose (>7.8 mmol/l 2 hrs after 75g OGTT). Subjects with IFG/IGT will have both impaired fasting glucose (5.6-7mmol/l), and impaired glucose tolerance (>7.8 mmol/l 2 hrs after 75g OGTT). Exclusion criteria include: regular exercise more than 2 hours/week, medications that could affect glucose, lipid metabolism, thyroid disease, a history of lung disease, pregnancy, active cigarette smoking or exposure to second hand smoke and severe plasma lipid disorders.

Metabolic study**:** Energy requirement was estimated based on DEXA determined fat free mass with the macronutrient intake at 55% carbohydrate, 15% protein, and 30% fat. Volunteers spent the night on the CTRC to ensure compliance with the overnight fast. The metabolic study consisted of a basal muscle biopsy followed by a 3-hour hyperinsulinemic euglycemic clamp at 40 mU/m^2/min for 3 hours. A variable infusion of 20% dextrose was infused to maintain blood glucose ~5 mmol/l. Standard Balke treadmill test was used to measure maximal work capacity (VO2max). Expired gases were collected and analyzed throughout the duration of the test at 20 sec intervals using standard indirect calorimetry (Parvo Medics Truemax 2400, Salt Lake City, UT). Heart rate and rhythm were monitored continually using a 12-lead ECG. The test was considered successful if oxygen consumption plateaued or the participant met three of the following four criteria: 1) maximum heart rate within 20 beats min-1 of the age-predicted maximal heart rate (220-age), 2) a RPE (Rating of Perceived Excertion) greater than 17, 3) respiratory exchange ratio greater than 1.10, and 4) volitional exhaustion by the participant. Maximum oxygen consumption was determined as the highest value observed during the test.

12 week intervention program**:** Everyone received a low-calorie diet consisting of a meal replacement product that can be consumed as a liquid or made into a variety of food forms (Health Nutrition Technology Inc., Carmel California). Subjects were provided powdered HealthOne formula and instructed to consume 5 portions per day providing 890 kcal/d, 75 g or protein, 15 g fat and 110 g of carbohydrate and 100% of the DRI of all vitamins, minerals and micronutrients. Subjects were allowed to consume non-caloric beverages but no other food intake was allowed. The delivery of this weight loss program has been supervised by the Clinical Core of the NORC (P30 DK048520) at the Anschutz Health and Wellness Center on the campus of the Anschutz Medical Center. Subjects were seen weekly in one-on-one sessions with a registered dietician for help with the diet and to receive nutritional counseling. Participants also underwent supervised endurance exercise training using well-described procedures used by the NORC energy balance core. Volunteers were asked to attend 4 sessions per week. Each session lasted 60 min and included a short warm-up of stretching exercises and walking, 40 to 50 min of endurance exercise, and a cool-down period. The exercise program consisted primarily of brisk walking or jogging, and was supplemented with rowing, stepping, or elliptical exercise to provide variety and relieve joint discomfort when necessary. Individualized exercise

prescriptions took into account the fitness level of the participant, preferences with regard to type of exercise, and any orthopedic limitations. The initial exercise prescription was 30 minutes at 65% of maximal HR, based on the highest HR attained during the baseline maximal exercise test, which will be repeated after 6 weeks of training. During the first 2 to 3 weeks of training, exercise duration and intensity was gradually increased to 45 min at 80 to 85% of maximal HR. The exercise prescription, which was updated every 2 weeks, was carefully geared to the participant's exercise capacity. The rate at which the intensity of the exercise is increased was determined by a) the magnitude of the training-induced increases in exercise capacity, and b) the subject's reaction to the exercise in terms of fatigue and musculoskeletal symptoms. Participants wore a HR monitor that captures and stores HR throughout the exercise session. Research technicians downloaded HR data after each session and entered the actual exercise performed into the study database.

2 week weight maintenance diet**:** Three servings of meal replacement per day along with one meal of typical foods for one additional month to stabilize weight. This period of the dietary intervention was supervised by research dieticians who have extensive experience helping subjects maintain a reduced state for metabolic studies at the University of Colorado CTRC.

Model and Statistics

Selection of Multivariate Regression hits: Selection of gene specific regression models for predicting FG and GIR was done based on a combination of model log-likelihood and negative log-likelihood. Log likelihood was estimated with the *mvregress* Matlab function. Additionally, we calculated the negative log-likelihood for multivariate regressions using Matlab's *mvregresslike* function for each model. Log-likelihood and negative log-likelihood distributions did not reveal a distinct cutoff criteria for model selection (ESM Fig. 1c). We thus manually applied a linear cutoff function to select a meaningful number of gene related models with high evaluation scores for both likelihood distributions (ESM Fig. S1c).

Transcriptomic profiling

Total RNA was isolated using RNeasy Kit (Qiagen, Hilden, Germany) according to manufacturer's instructions. For library preparation, 1 µg of total RNA per sample was used. RNA molecules were poly(A) selected, fragmented, and reverse transcribed with the Elute, Prime, Fragment Mix (EPF, Illumina). End repair, A-tailing, adaptor ligation, and library enrichment were performed as described in the TruSeq Stranded mRNA Sample Preparation Guide (Illumina). RNA libraries were assessed for quality and quantity with the Agilent 2100 BioAnalyzer and the Quant-iT Pico- Green dsDNA Assay Kit (Life Technologies). IMAT strand-specific RNA libraries were sequenced as 100+100 bp paired-end runs on an Illumina HiSeq2500 platform and muscle strand-specific RNA libraries were sequenced as 100+100 bp paired-end runs on an Illumina HiSeq4000 platform. The STAR aligner* (v 2.4.2a) with modified parameter settings (–twopassMode = Basic) was used for split-read alignment against the human genome assembly hg19 (GRCh37) and UCSC known Gene annotation. To quantify the number of reads mapping to annotated genes we used HTseq-count° (v0.6.1). RNA-Seq count files filtered for genes with an average read count above 50 counts per sample. DESeq2 [1] was used for expression level normalization using variance stabilizing transformation.

Gene expression analysis.

Total RNA was isolated using RNeasy Kit (Qiagen, Hilden, Germany) according to manufacturer's instructions. cDNA synthesis was performed with QuantiTect Reverse Transcription Kit (Qiagen, Hilden, Germany) according to manufacturer's instructions. Muscle gene expression was profiled with quantitative real-time RT–PCR using SYBR Green with validated primers. The relative expression of the selected genes was normalized to the reference gene TATA-Box Binding-Protein (*TBP*) as fold change to control. A full list of primers is given in ESM Table 2.

ESM References

[1] Love, M.I., W. Huber, and S. Anders, *Moderated estimation of fold change and dispersion for RNA-seq data with DESeq2.* Genome Biol, 2014. **15**(12): p. 550.

ESM Figures

ESM Fig. 1

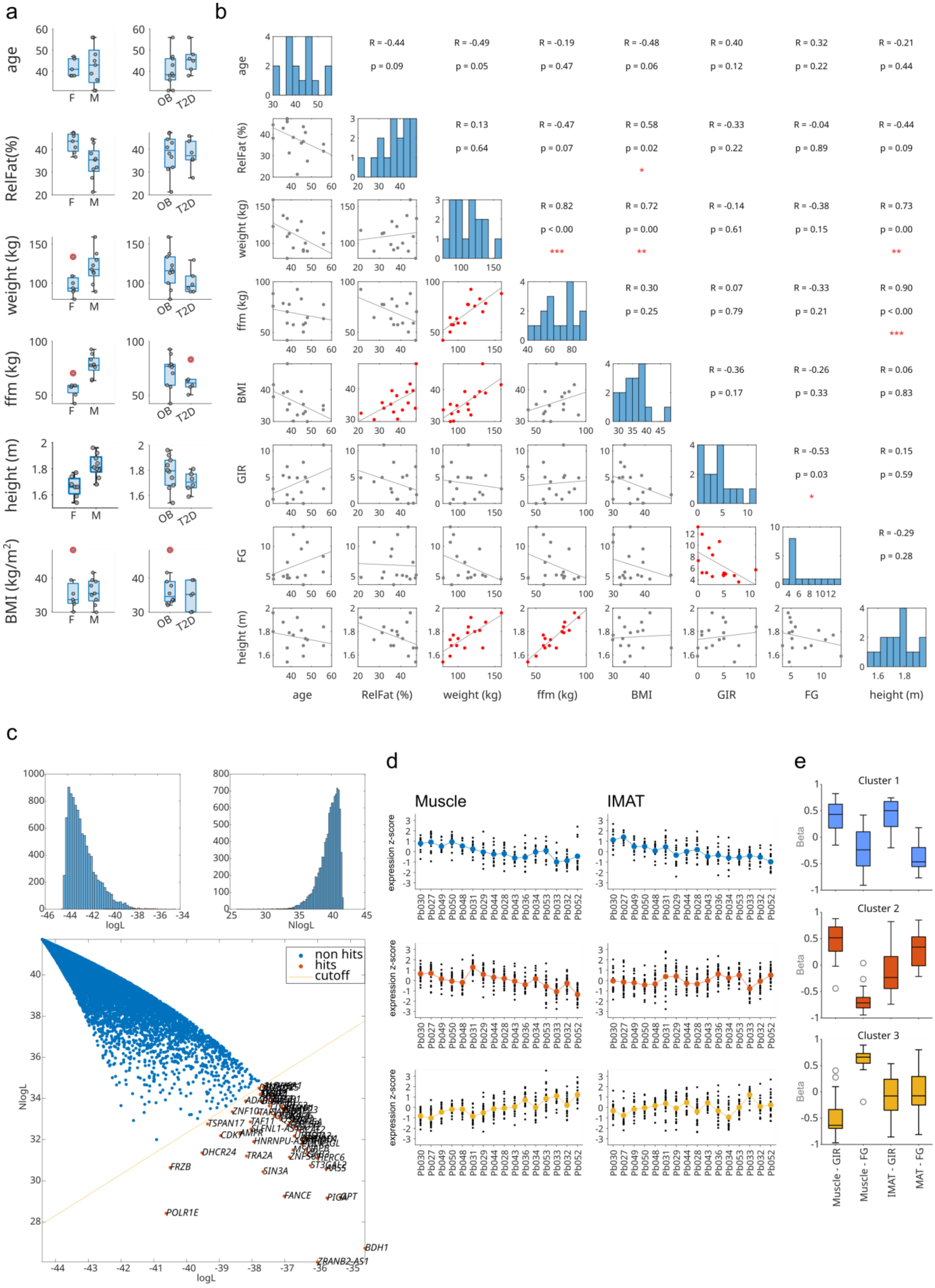

ESM Fig. 1

a) Boxplot comparing clinical parameter distributions between subjects of different classifications and gender. OB: obese, T2D: type 2 diabetic F: female, M: male. Red marked dots indicate outliers.

b) Correlation plot for pairwise comparisons between measured clinical parameter including histograms showing parameter distribution. Red dots indicate significant correlation. Upper right triangle lists correlation coefficients and p-values.

c) Distribution of mRNA wise estimated regression model log-Likelihood (top left) and Negative log-likelihood (top right) and scattered versus each other (bottom). Yellow cutoff line indicates selection function, red dots refer to mRNAs selected for further investigation.

d) Box plots show distribution of the four regression coefficients for each cluster. Horizontal lines refer to median, boxes to upper and lower quartile and whiskers to maxima and minima of the distributions. Dots denote outliers.

e) Subject wise normalized mRNA expression for the three gene clusters for Muscle (left) and IMAT (tissue).

ESM Fig. 2

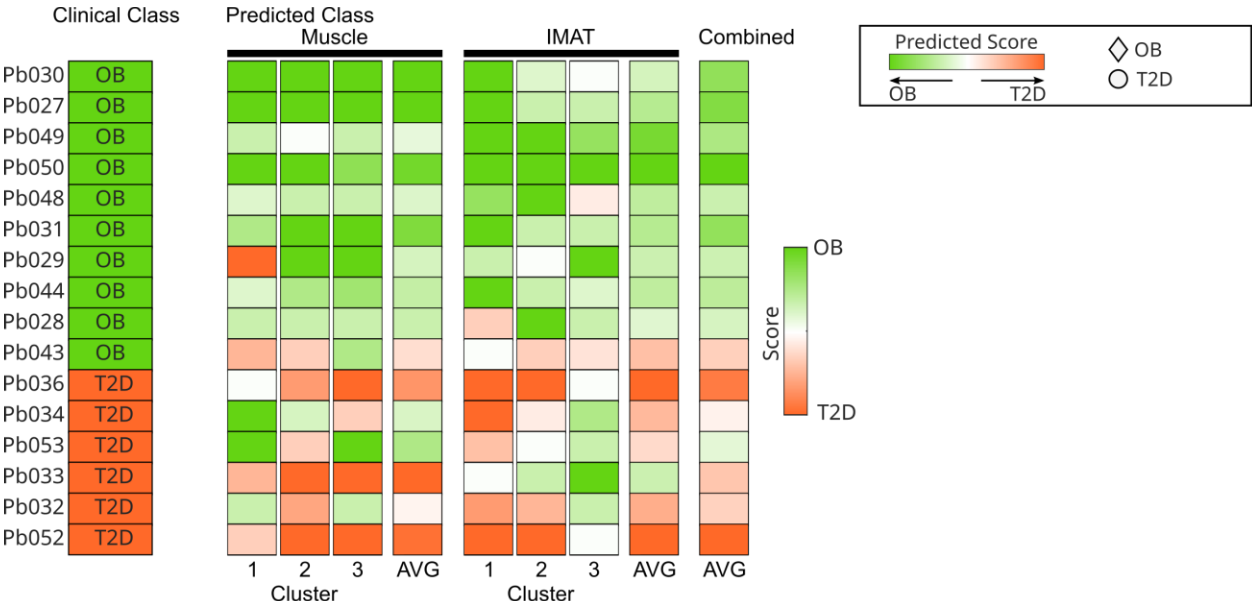

ESM Fig. 2

Estimated classification scores by k-nearest neighbor classification for each cluster and tissue including average scores for each tissue and for both tissues combined.

ESM Fig. 3

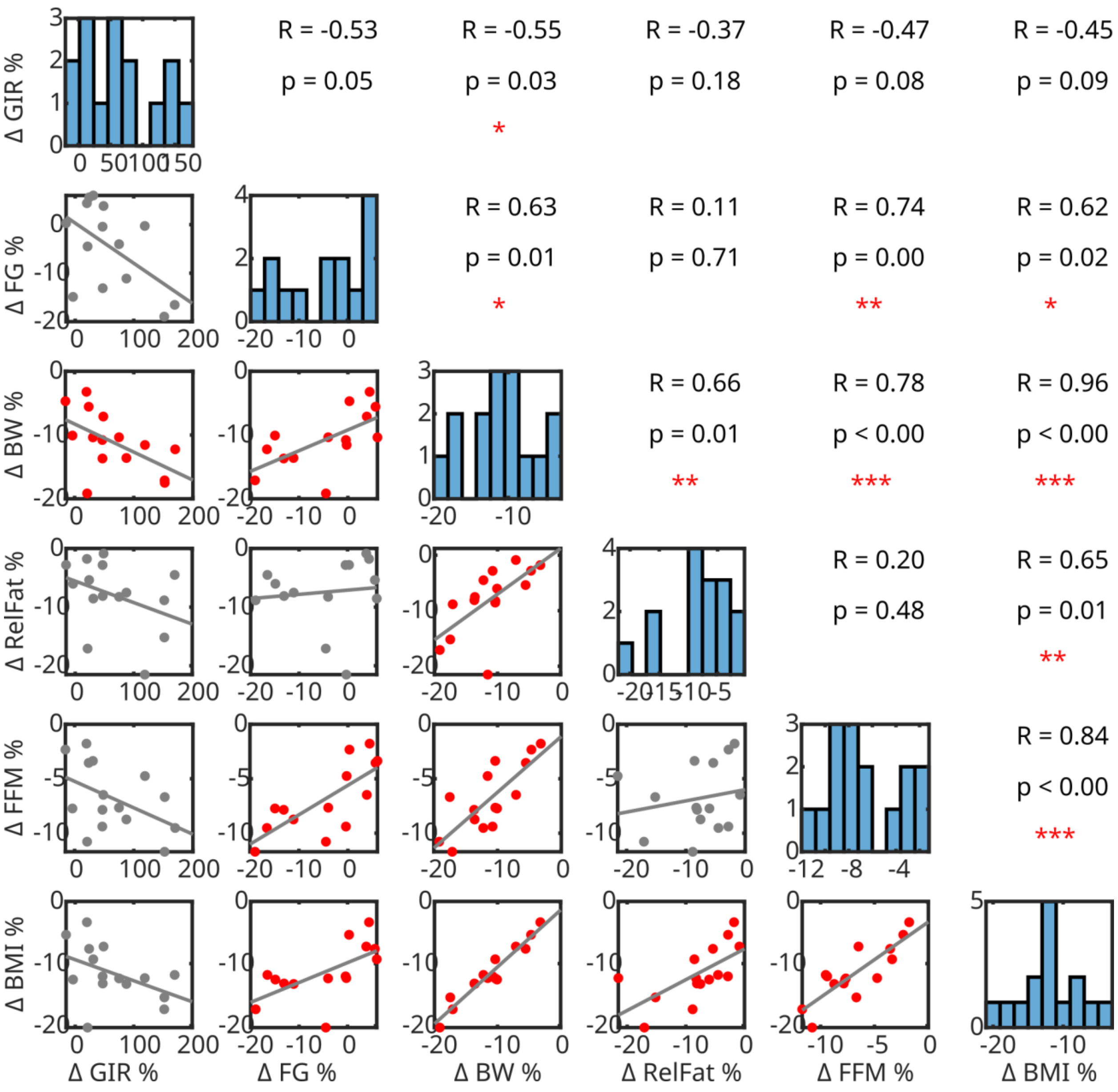

ESM Fig. 3

Correlation plot for pairwise comparisons between with relative change (pre to post) of clinical parameter including histograms showing parameter distribution. Red dots indicate significant correlation. Upper right triangle lists correlation coefficients and p-values.

ESM Fig. 4

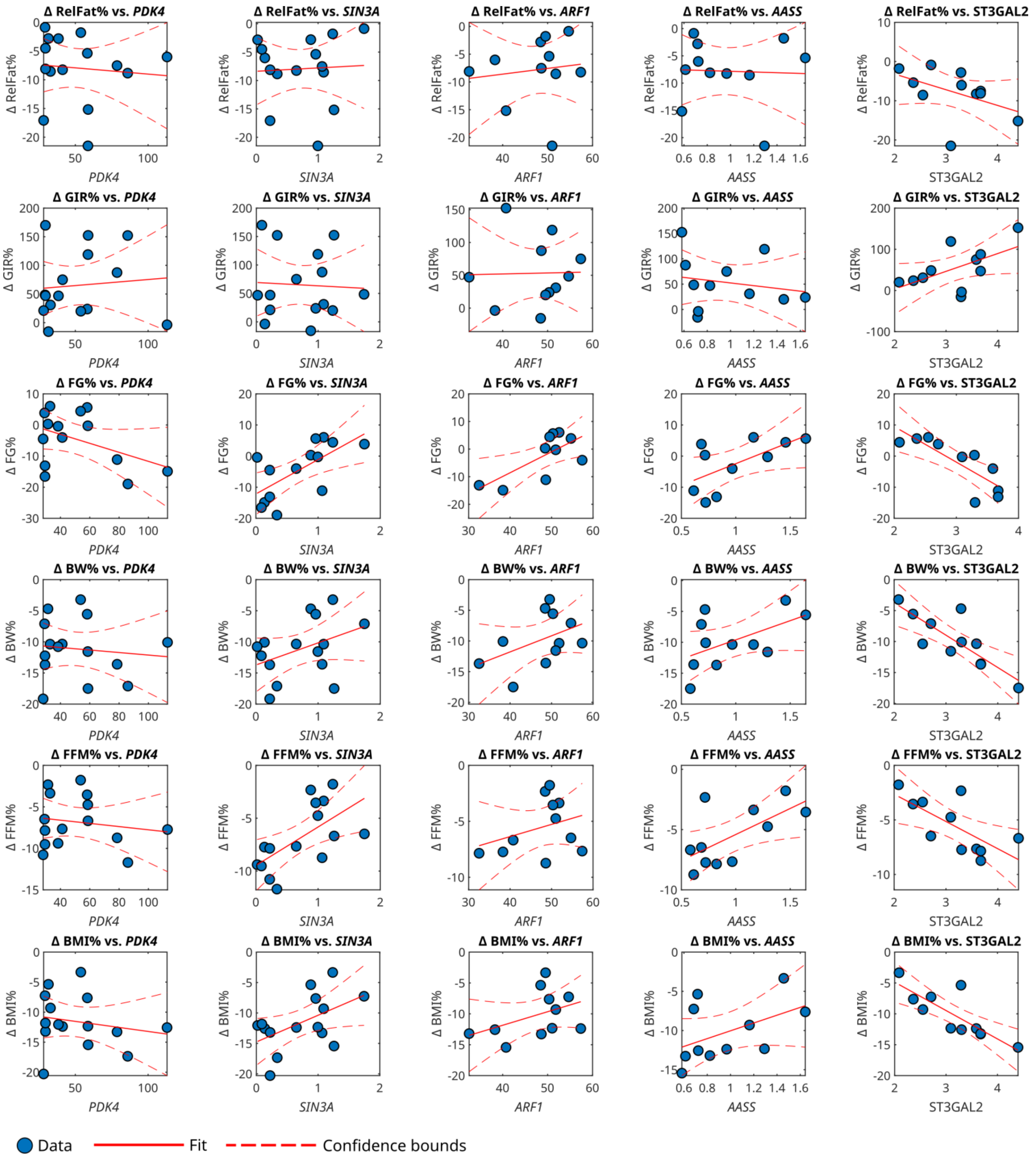

ESM Fig. 4

Correlation plots for five selected mRNAs with clinical parameters. Red lines refer to estimated correlation, dashed lines to confidence intervals.

Supplementary Tables

ESM Table 1

Symbol	logL	NlogL	β muscle - GIR	β muscle - FG	β IMAT - GIR	β IMAT - FG	Clus-ter
AASS	-35.75	30.59	0.77	-0.88	0.01	0.35	2
ADAR	-38.20	33.88	0.71	-0.45	0.23	-0.02	2
ALDH6A1	-37.65	34.59	0.21	-0.23	0.52	-0.46	1
AMFR	-38.38	32.30	-0.33	0.82	-0.12	-0.09	3
ARF1	-36.85	33.08	-0.69	0.76	0.46	-0.48	3
BCAT2	-36.57	32.46	0.63	-0.44	0.20	-0.47	1
BDH1	-34.56	26.73	0.68	-0.30	0.22	-0.56	1
C7orf25	-37.59	34.51	-0.66	0.64	0.24	-0.22	3
CCDC176	-37.31	33.13	0.38	-0.77	0.41	0.18	1
CDK7	-38.96	32.19	0.28	-0.77	-0.30	-0.02	2
CLEC3B	-36.91	32.72	0.61	-0.80	0.21	-0.21	2
DBNDD1	-36.50	31.99	-0.68	0.89	0.05	-0.26	3
DDR1	-37.72	33.85	0.83	-0.02	-0.20	-0.65	1
DHCR24	-39.53	31.36	-0.01	0.34	0.73	-0.17	1
DHTKD1	-37.79	34.50	0.61	-0.59	-0.03	-0.19	1
ENPP1	-37.74	34.19	0.73	-0.54	-0.13	0.09	1
FANCE	-37.02	29.26	0.55	0.10	0.54	-0.54	1
FRZB	-40.50	30.65	0.10	0.52	-0.08	0.38	3
GAB3	-36.33	31.26	0.63	-0.81	-0.04	-0.14	2
GNG5	-37.68	34.24	-0.41	0.62	-0.49	0.16	3
GPT	-35.31	29.21	0.17	0.00	0.68	-0.77	1
HERC6	-36.02	31.10	0.70	-0.72	0.39	-0.01	2
HFE2	-36.76	32.91	-0.70	0.73	0.03	0.02	3
HNRNPU-AS1	-37.95	31.90	0.41	-0.91	-0.36	0.39	2
HSPA2	-36.40	32.15	-0.73	0.76	0.05	-0.06	3
LDHD	-36.46	31.89	0.49	-0.24	0.49	-0.53	1
MSL3	-36.75	31.50	0.28	0.42	-0.79	0.43	3
NAPB	-36.35	31.55	0.53	-0.55	-0.71	0.46	2
NEGR1	-37.38	33.94	0.73	-0.80	-0.46	0.68	2
PDK4	-37.19	33.15	-0.97	0.60	0.27	-0.01	3
PIGA	-35.72	29.17	0.87	-0.60	-0.14	0.55	2
PITHD1	-37.07	33.50	-0.39	0.55	-0.40	0.29	3
POLR1E	-40.62	28.42	-0.44	0.04	0.82	0.14	2
POLR3GL	-36.49	31.79	-0.61	0.80	-0.16	-0.25	3
PPM1K	-36.34	32.01	0.85	-0.83	-0.23	0.23	2
QSOX2	-37.13	32.97	0.88	-0.71	-0.25	0.49	2
RCAN1	-37.06	33.50	0.40	-0.19	-0.86	0.80	3
SAYSD1	-37.42	33.32	-0.32	0.46	-0.28	0.40	3
SF3A1	-37.51	33.96	-0.15	0.35	0.75	-0.56	1
SIN3A	-37.67	30.44	-0.02	-0.64	0.70	-0.01	2
SLFNL1-AS1	-38.03	32.51	0.06	0.42	0.73	-0.45	1

SNAP23	-36.99	33.42	0.76	-0.75	-0.37	0.34	2
SPCS2	-37.10	33.63	-0.69	0.68	-0.08	0.09	3
SSBP2	-37.75	34.21	-0.37	0.42	-0.39	0.59	3
SSU72	-36.71	32.58	-0.69	0.70	-0.13	-0.25	3
ST3GAL2	-36.22	30.74	-0.91	0.67	0.30	-0.43	3
SYAP1	-37.40	33.83	-0.86	0.65	0.54	-0.62	3
TAF1	-37.81	33.30	0.25	-0.71	0.47	-0.05	2
TAF11	-38.05	32.86	0.33	-0.94	-0.59	0.85	2
TIGD7	-37.75	34.19	0.03	-0.27	-0.74	0.54	2
TRA2A	-38.17	31.20	0.13	-0.70	-0.38	0.53	2
TSPAN17	-39.36	32.76	-0.21	0.70	-0.35	-0.15	3
TTC7B	-37.48	33.64	-0.59	0.68	-0.05	-0.24	3
UBTD1	-36.72	32.18	-0.67	0.65	0.47	-0.81	3
ZNF10	-38.60	33.36	0.38	-0.63	-0.12	0.54	2
ZNF561	-36.86	31.15	0.52	-0.85	-0.23	0.23	2
ZNF692	-37.66	34.56	0.23	-0.40	-0.72	0.77	2
ZNF75A	-36.84	32.83	0.36	-0.66	-0.47	0.21	2
ZRANB2-AS1	-36.01	26.06	0.33	-0.91	0.59	-0.32	1

ESM Table 1

Top 59 Genes with high associations to GIR and FG. Log-likelihood, neg-log-Likelihood, β values and Clustering coefficients are given. Genes selected for qPCR validation with intervention cohort are color shaded.

ESM Table 2

AASS forward	5’-GAATCGGCGGGCCATTCAT-3‘,
AASS reverse	5’-CTGAGCTTTTATTGTGTGGGAGA-3’;
ARF1 forward	5’-ATGGGGAACATCTTCGCCAAC-3‘,
ARF1 reverse	5’-GTGGTCACGATCTCACCCAG-3’;
BCAT2 forward	5’-GCTCAACATGGACCGGATG-3‘,
BCAT2 reverse	5’-CCGCACATAGAGGCTGGTG-3’;
DBNDD1 forward	5’-TCTTTGCTGACTCGGACGAC-3‘,
DBNDD1 reverse	5’-CCACAGTGAGAAACGTGTCCA-3’;
LDHD forward	5’-CCGTAGCCCGCATTGAGTT-3‘,
LDHD reverse	5’-CTGCTGGACTATCTCCTCTGT-3’;
NAPB forward	5’-CAGGAAACGCATTTTGTCAGG-3‘,
NAPB reverse	5’-GGGATCTGCCTTTTTGTAAGCAT-3’;
PDK4 forward	5’-GGAAGCATTGATCCTAACTGTGA-3‘,
PDK4 reverse	5’-GGTGAGAAGGAACATACACGATG-3’;
PIGA forward	5’-GTTGGCAGTTTTCAACTTCCTCT-3‘,
PIGA reverse	5’-TGGCCCAGTGGCATCTATTG-3’;
POLR3GL forward	5’-ATCAGATGTCAGGTCCGATTGA-3‘,
POLR3GL reverse	5’-AGCAGAATTGTAATCCGTTCCTT-3’;
SIN3A forward	5’-ATGAGTCTCTGGAAAGTACGAGG-3‘,
SIN3A reverse	5’-GCATCTGGTAGGAATTGTCCAAA-3’;
SNAP23 forward	5’-CCTGTGGAGTTTAATCATGCCA-3‘,
SNAP23 reverse	5’- CCACAGCATTTGTTGAGTTCTG -3’;
SPCS2 forward	5’-CGTAGCGGCTTGTTGGATAAG-3‘,
SPCS2 reverse	5’-GTGAGGCGACCATCAATTAGAC-3’;
SSU72 forward	5’-TCCCGACAAGCCCAATGTTTA-3‘,
SSU72 reverse	5’-CTCTTCGCAAGTGAGGATCAG-3’;
ST3GAL2 forward	5’-CGTCTGGACCCGAGAGAAC-3‘,
ST3GAL2 reverse	5’-GCCAGGCACTATCTGGAACA-3’;
TBP forward	5’-AACAACAGCCTGCCACCTTA-3‘,
TBP reverse	5’-GCCATAAGGCATCATTGGAC-3’;
UBTD1 forward	5’-CGGAGCAAACGGGATGAGTT-3‘,
UBTD1 reverse	5’-GCGGTGACAGGCAGTAGAT-3’.

ESM Table 2

List of all used primers for qPCR expression measurements. TBP was used as a house keeping gene reference to estimate relative gene expression.

ESM Table 3

GENE	P RELFAT%	PADJ RELFAT%	COEF RELFAT%	R2 RELFAT%	P GIR%	PADJ GIR%	COEF GIR%	R2 GIR%	P FG%	PADJ FG%	COEF FG%	R2 FG%	P BW%	PADJ BW%	COEF BW%	R2 BW%	P FFM%	PADJ FFM%	COEF FFM%	R2 FFM%	P BMI%	PADJ BMI%	COEF BMI%	R2 BMI%
**LDHD**	0.495	0.928	-2.23	0.037	0.284	0.949	-34.66	0.088	0.735	0.809	1.67	0.010	0.447	0.696	-1.98	0.045	0.523	0.944	-1.09	0.032	0.298	0.615	-2.54	0.083
**UBTD1**	0.701	0.944	-2.11	0.012	0.866	0.949	-9.34	0.002	0.433	0.590	6.99	0.052	0.851	0.851	0.82	0.003	0.259	0.647	3.17	0.097	0.536	0.615	2.56	0.030
**PDK4**	0.741	0.944	-0.02	0.009	0.748	0.949	0.21	0.008	0.124	0.310	-0.15	0.186	0.699	0.749	-0.02	0.012	0.573	0.944	-0.02	0.025	0.498	0.615	-0.03	0.036
**DBNDD1**	0.400	0.858	-0.28	0.055	0.180	0.949	4.38	0.134	0.755	0.809	-0.21	0.008	0.510	0.696	-0.18	0.034	0.885	0.944	0.03	0.002	0.723	0.723	-0.09	0.010
**SIN3A**	0.851	0.944	0.59	0.003	0.853	0.949	-5.87	0.003	**0.010**	0.074	11.05	0.439	0.141	0.414	3.55	0.159	**0.013**	0.140	3.64	0.390	**0.047**	0.329	4.38	0.270
**SPCS2**	0.385	0.858	1.01	0.085	0.380	0.949	-8.70	0.087	0.958	0.958	-0.12	0.000	0.470	0.696	0.61	0.060	0.748	0.944	-0.16	0.012	0.487	0.615	0.52	0.055
**ARF1**	0.711	0.944	0.10	0.016	0.949	0.949	0.15	0.000	**0.024**	0.120	0.76	0.491	0.166	0.414	0.26	0.202	0.326	0.698	0.11	0.107	0.194	0.485	0.22	0.180
**BCAT2**	0.393	0.858	25.17	0.082	0.423	0.949	-202.04	0.073	0.054	0.162	75.96	0.389	0.051	0.382	37.97	0.360	0.127	0.381	17.64	0.239	0.066	0.329	32.11	0.328
**NAPB**	0.170	0.858	8.98	0.198	0.902	0.949	-7.29	0.002	0.413	0.590	7.56	0.085	0.560	0.700	2.87	0.039	0.800	0.944	-0.72	0.007	0.473	0.615	3.12	0.059
**PIGA**	0.224	0.858	86.00	0.160	0.724	0.949	-221.11	0.014	0.317	0.590	100.37	0.125	0.126	0.414	75.90	0.240	0.695	0.944	11.82	0.018	0.137	0.421	65.73	0.229
**AASS**	0.908	0.944	-0.65	0.002	0.570	0.949	-27.05	0.037	0.054	0.162	13.91	0.390	0.097	0.414	6.21	0.275	**0.034**	0.169	4.32	0.410	0.140	0.421	4.98	0.225
**ST3GAL2**	0.161	0.858	-3.99	0.206	0.066	0.949	43.03	0.327	**0.004**	0.061	-11.45	0.664	**0.002**	**0.028**	-5.22	0.677	**0.019**	0.140	-2.50	0.477	**0.002**	**0.032**	-4.60	0.668
**POLR3GL**	0.944	0.944	-0.19	0.001	0.196	0.949	23.40	0.200	0.526	0.657	-2.32	0.052	0.241	0.516	-1.90	0.167	0.115	0.381	-1.72	0.281	0.416	0.615	-1.32	0.084
**SSU72**	0.774	0.944	0.22	0.010	0.597	0.949	3.38	0.032	0.355	0.590	0.97	0.107	0.615	0.709	0.27	0.029	0.944	0.944	0.02	0.001	0.574	0.615	0.27	0.036
**SNAP23**	0.389	0.858	1.46	0.084	0.641	0.949	-6.81	0.025	0.236	0.506	3.45	0.170	0.320	0.601	1.20	0.109	0.855	0.944	0.13	0.004	0.334	0.615	1.04	0.104

ESM Table 3

Statistics of regression models predicting relative change of clinical parameters from pre intervention gene expression. P-values, adjusted p-values, coefficients and R^2 are summarized for each gene and model.

[1] Love MI, Huber W, Anders S (2014) Moderated estimation of fold change and dispersion for RNA-seq data with DESeq2. Genome Biol 15(12): 550. 10.1186/s13059-014-0550-8
